# Structural and functional analyses of the echinomycin resistance conferring protein Ecm16 from *Streptomyces lasalocidi*

**DOI:** 10.1038/s41598-023-34437-9

**Published:** 2023-05-17

**Authors:** Priyanka Gade, Amanda Erlandson, Anwar Ullah, Xi Chen, Irimpan I. Mathews, Paola E. Mera, Chu-Young Kim

**Affiliations:** 1 Department of Chemistry and Biochemistry, The University of Texas at El Paso, El Paso, TX USA; 2 Department of Microbiology, School of Molecular and Cellular Biology, University of Illinois Urbana-Champaign, Urbana, IL USA; 3 Key Laboratory of Synthetic and Natural Functional Molecule of the Ministry of Education, College of Chemistry and Materials Science, Northwest University, Xi’an, 710127 China; 4 Stanford Synchrotron Radiation Lightsource, SLAC National Accelerator Laboratory, Menlo Park, CA USA; 5 Present Address: Department of Biochemistry, School of Molecular and Cellular Biology, University of Illinois Urbana-Champaign, Urbana, IL USA

**Keywords:** Biochemistry, Microbiology, Structural biology

## Abstract

Echinomycin is a natural product DNA bisintercalator antibiotic. The echinomycin biosynthetic gene cluster in *Streptomyces lasalocidi* includes a gene encoding the self-resistance protein Ecm16. Here, we present the 2.0 Å resolution crystal structure of Ecm16 bound to adenosine diphosphate. The structure of Ecm16 closely resembles that of UvrA, the DNA damage sensor component of the prokaryotic nucleotide excision repair system, but Ecm16 lacks the UvrB-binding domain and its associated zinc-binding module found in UvrA. Mutagenesis study revealed that the insertion domain of Ecm16 is required for DNA binding. Furthermore, the specific amino acid sequence of the insertion domain allows Ecm16 to distinguish echinomycin-bound DNA from normal DNA and link substrate binding to ATP hydrolysis activity. Expression of *ecm16* in the heterologous host *Brevibacillus choshinensis* conferred resistance against echinomycin and other quinomycin antibiotics, including thiocoraline, quinaldopeptin, and sandramycin. Our study provides new insight into how the producers of DNA bisintercalator antibiotics fend off the toxic compounds that they produce.

## Introduction

Quinomycin antibiotics act by noncovalently binding to the DNA double helix^1^. They are DNA bisintercalators, compounds that bind reversibly to the DNA duplex by inserting a pair of aromatic ring groups in between adjacent base pairs of the DNA. There has been much interest in this family of compounds due to their potent antimicrobial and antitumor activity, culminating in a large body of biochemical, structural, and clinical knowledge^2^. However, mechanistic details about bacterial resistance against DNA bisintercalators remains limited. While no resistance element against these compounds has been discovered so far in pathogenic bacteria, it is prudent to identify and elucidate resistance mechanisms that are present in the natural environment. Knowledge of basic resistance mechanisms will allow researchers to develop new therapeutic strategies before quinomycin resistance becomes a clinical problem.

Quinomycin antibiotic producers typically contain one or more self-resistance genes, which are found either within the antibiotic biosynthetic gene cluster or elsewhere in the genome. For example, the triostin producing bacterium contains a gene that encodes for an ABC transporter^3^, and the thiocoraline producer contains genes that encode for an ABC transporter as well as a thiocoraline sequestration protein^4,5^, Interestingly, quinomycin producers also contain a gene for a UvrA-like protein in their biosynthetic gene cluster (Table 1). UvrA is a DNA repair enzyme from the universal prokaryotic nucleotide excision repair (NER) pathway. Briefly, UvrA recognizes the DNA damage and recruits UvrB to the lesion site. UvrB recruits UvrC which cleaves the phosphodiester bond eight nucleotides upstream and up to five nucleotides downstream of the modified nucleotide. Next, UvrC recruits the UvrD helicase which displaces the cleaved DNA fragment. DNA polymerase I synthesizes the missing stretch of DNA using the undamaged complementary strand as a template and DNA ligase seals the breaks, thus completing the repair^6^.

**Table 1:** UvrA-like proteins expressed by producers of quinomycin antibiotics.

Protein	Number of residues	Species	Confers resistance against^a^	Sequence identity to Ecm16
Ecm16	792	*Streptomyces lasaliensis*	Echinomycin	–
AcmrC	756	*Streptomyces anulatus*	Actinomycin D	52.1%
CmrX	826	*Streptomyces griseus*	Chromomycin A_3_	37.8%
DrrC	764	*Streptomyces peucetius*	Doxorubicin	51.2%
MtrX	828	*Streptomyces argillaceus*	Mithramycin	34.8%
Swb15	792	*Streptomyces* sp. SNA15896	SW-163	79.8%
TioU	874	*Micromonospora* sp. ML1	Thiocoraline	71.2%
TrsM	792	*Streptomyces triostinicus*	Triostin A	87.8%

^a^Resistance activity of Ecm16 and DrrC have been experimentally confirmed. Others are predictions based on sequence similarity and the natural product biosynthetic gene cluster that the encoding gene is found in.

Echinomycin is a prototypical DNA bisintercalator produced by multiple actinomycetes, including *Streptomyces echinatus* and *Streptomyces lasalocidi* (formerly known as *S. lasaliensis*)^7–9^. It is a cyclic depsipeptide that contains two quinoxaline groups and an unusual thioacetal bridge (Fig. 3)^10^. Echinomycin shows potent antimicrobial activity against methicillin-resistant *Staphylococcus aureus* and vancomycin-resistant enterococci^11,12^ but it is not used clinically due to solubility and toxicity issues. The echinomycin biosynthetic gene cluster from *S. lasalocidi* contains genes that encode for enzymes that synthesize the quinoxaline group, enzymes that construct the peptide backbone, and genes that encode for proteins with unknown function^13^. One of the functionally uncharacterized proteins is Ecm16, which was postulated to provide self-protection against echinomycin based on the sequence identity (~ 30%) it shares with the prokaryotic UvrA proteins that function in the NER pathway^13^.

We have previously reported the in vivo and in vitro functional characterization of Ecm16^14^. The main findings of that study are (1) the echinomycin sensitive *Escherichia coli* K12 becomes echinomycin resistant upon transformation with the *ecm16* encoding plasmid, (2) Ecm16 does not require participation of the NER proteins UvrA, UvrB, UvrC, or UvrD to provide echinomycin resistance, (3) Ecm16 does not complement UvrA function, (4) Ecm16’s ATPase activity is essential for its anti-echinomycin activity, (5) Ecm16 binds to double-stranded DNA in a nucleotide sequence independent manner, and (6) Ecm16 binds to echinomycin-containing DNA ~ two-fold more strongly than to echinomycin-free DNA. In the current study, we have determined the crystal structure of Ecm16 to provide a structural context to its function. We have also performed mutational studies to dissect the role of Ecm16’s insertion domain. In UvrA proteins, the insertion domain is involved in damage-specific DNA binding^15^. Lastly, we have probed the substrate specificity of Ecm16 by challenging *ecm16*-expressing cells with a series of quinomycin and non-quinomycin DNA targeting antibiotics.

## Results

### X-ray crystal structure of Ecm16

**Figure 1:**
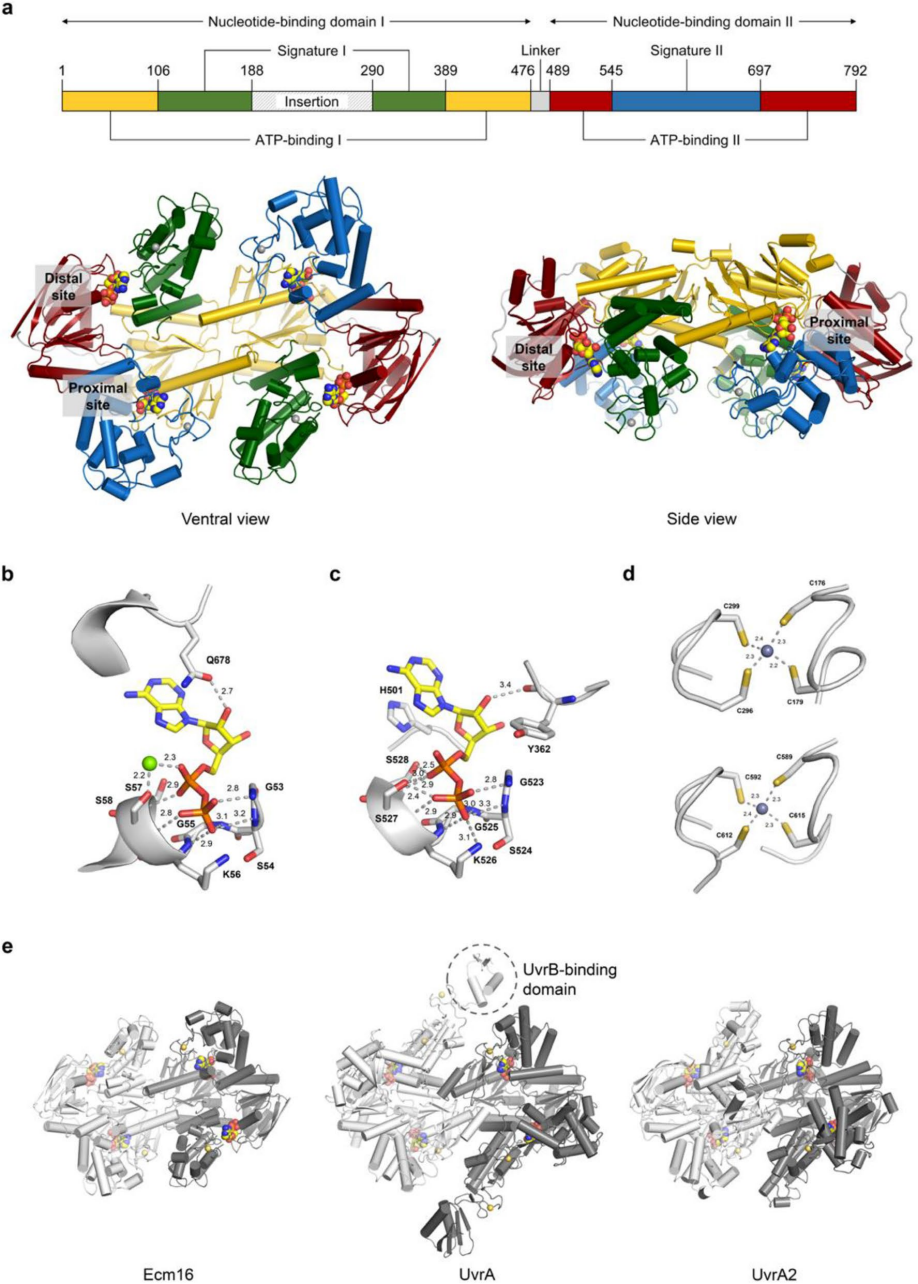
Crystal structure of Ecm16. (**a**) Domain organization and overall structure of the Ecm16 homodimer. Insertion domain is present in the primary structure, but it is not observed in the crystal structure. ADP, Zn^2+^, and Mg^2+^ atoms are drawn as spheres (C: yellow, N: blue, O: red, P: orange, Zn: grey, Mg: green). (**b**) Proximal nucleotide-binding site. Atom-to-atom distances are given in Å. (**c**) Distal nucleotide-binding site. (**d**) Top: zinc-binding module 2. Bottom: zinc-binding module 3. (**e**) Crystal structure of Ecm16 from *Streptomyces lasalocidi* (PDB ID: 7SH1), UvrA from *Bacillus stearothermophilus* (PDB ID: 2R6F), and UvrA2 from *Deinococcus radiodurans* (PDB ID: 2VF8).

We have determined the structure of Ecm16 bound to adenosine diphosphate (ADP) at 2.0 Å resolution (Fig. 1a). Our final model consists of the Ecm16 homodimer, four ADP, two Mg^2+^, four Zn^2+^, and 612 water molecules (Table 2). Some residues, including 183-293 of chain A and 185-295 of chain B that includes the entire insertion domain, were not modelled due to missing electron density (Supplementary Table 1). Each protomer of Ecm16 contains two ABC ATPase motifs, referred to as nucleotide-binding domain I and II (NBD-I and NBD-II). NBD-I consists of the ATP-binding I domain, signature I domain, and insertion domain. The insertion domain was not visible in the crystal structure, presumably because it is disordered. NBD-II consists of the ATP-binding II domain and the signature II domain. NBD-II is missing the helix-turn-strand that corresponds to residue 66-99 of NBD-I (Supplementary Fig. 1). Other than these two differences, NBD-I and NBD-II have a relatively similar overall structure (RMSD = 1.5 Å for 208 C_α_ atoms). The dimer interface of Ecm16 buries ~ 3900 Å^2^ of surface area and is comprised of residues from the ATP-binding I, signature I, and signature II domains (Supplementary Fig. 2). The ventral side of Ecm16 features an extended groove that is lined with numerous basic residues (K136, K143, K381, R384, R567, K568, R537, K549, K572, K577) (Supplementary Fig. 3). This ~ 10 nm long and ~ 2 nm wide groove can potentially accommodate a ~ 32 bp B-form DNA and provides a structural basis for the previously reported DNA-binding activity of Ecm16^14^.

**Table 2:** Data processing and refinement statistics.

Parameter	Ecm16
Data collection	
Beamline	APS 17-ID-B
Processing software	XDS
Wavelength (Å)	1.0
Resolution range (Å)	36.88–2.04 (2.11–2.04)^a^
Space group	*P*3_1_21
Unit cell a, b, c (Å)	141.1, 141.1, 173.6
Unit cell α, β, γ (°)	90, 90, 120
Total reflections	2,590,370 (263,214)
Unique reflections	126,891 (12,542)
Multiplicity	20.4 (20.7)
Completeness (%)	99.96 (99.99)
Mean *I/sigma(I)*	23.54 (2.30)
Wilson B factor (Å^2^)	38.46
* R-meas*	0.09 (1.56)
CC_1/2_	1 (0.79)
Refinement	
Reflections used in refinement	126,908 (12,535)
Reflections used for *R*_free_	6150 (591)
* R*_work_	0.20 (0.30)
* R*_free_	0.24 (0.35)
No. of nonhydrogen atoms	10,007
Macromolecules	9276
Ligands	119
Solvent	612
Protein residues	1252
RMS^b^	
Bond length (Å)	0.02
Bond angle (°)	1.96
Ramachandran favored (%)	96.91
Ramachandran allowed (%)	2.93
Ramachandran outliers (%)	0.16
Rotamer outliers (%)	3.32
Clash score	3.46
Average B factor (Å^2^)	47.13
Macromolecules	47.23
Ligands	38.47
Solvent	47.19
Number of TLS groups	2
PDB ID	7SH1

^a^Values in parentheses are for the highest-resolution shell.

^b^RMS, root mean square.

Ecm16 has a total of four nucleotide-binding sites, two proximal and two distal nucleotide-binding sites (Fig. 1a). The proximal nucleotide-binding site is located ~ 19 Å from the Ecm16 dimer interface and it is sandwiched between ATP-binding domain I and signature domain II. The distal nucleotide-binding site is located ~ 26 Å from the dimer interface and it is sandwiched between ATP-binding domain II and signature domain I. Each ATP-binding domain contains a Walker A motif, Walker B motif, and the α-helical ABC signature subdomain containing the LSGGQ sequence typically found in ABC transporters^16^ and DNA repair proteins^17^. The ATP-binding and signature domains are connected by the Q-loop, which is the site of major conformational change and coupling of energy converting domains of NBD on other ATPases^18^. Electron density at both the proximal and distal nucleotide-binding sites showed the presence of ADP (Supplementary Fig. 4). To further confirm the identity of the nucleotide bound to Ecm16, we performed liquid chromatography analysis of the protein extract. Only ADP was detected in this experiment (Supplementary Fig. 5), indicating that the nucleotide observed in the Ecm16 crystal structure is ADP, and not ATP.

Mg^2+^ ions are observed only at the two proximal nucleotide-binding sites, and not at the distal sites, even though Ecm16 was crystallized in the presence of 10 mM MgCl_2_ (Fig. 1b, c). The reason for this is not clear but a similar observation was made for the crystal structure of UvrA from *Bacillus stearothermophilus*^19^. The phosphate groups of ADP participate in an extensive hydrogen bond network involving the residues of the Walker A motif, while the ribose sugar and adenine base form relatively few interactions with Ecm16. The conserved histidine residue at position 501 stacks well against the adenine ring of ADP at the distal site. Each Ecm16 protomer contains two zinc-binding modules, which correspond to the UvrA zinc-binding module 2 and 3 observed in all UvrA crystal structures reported so far. In module 2, Zn^2+^ is coordinated to C176, C179, C296, and C299, while in module 3, Zn^2+^ is coordinated to C589, C592, C612, and C615 (Fig. 1d). These zinc-coordinating residues are conserved in UvrA and UvrA2 proteins^14,15^.

The three-dimensional structure of Ecm16 resembles that of UvrA from *B. stearothermophilus* (RMSD = 2.6 Å for 1002 C_α_ atoms) and UvrA2 from *Deinococcus radiodurans* (RMSD = 1.7 Å for 933 C_α_ atoms) (Fig. 1e, Supplementary Fig. 6). The structural similarity of Ecm16, UvrA, and UvrA2 explains their common functionalities such as DNA binding and ATP hydrolysis^15,20^. However, Ecm16, like UvrA2, lacks the UvrB-binding domain and its associated zinc-binding module 1 which are found in all UvrA proteins, indicating that Ecm16 does not interact with UvrB from the NER pathway. This is consistent with our previous report that both the wild type and the UvrB knockout strains of *E. coli* K12 became resistant to echinomycin upon induction of expression of *ecm16* encoded in trans^14^.

### Insertion domain is required for DNA binding activity of Ecm16

**Figure 2:**
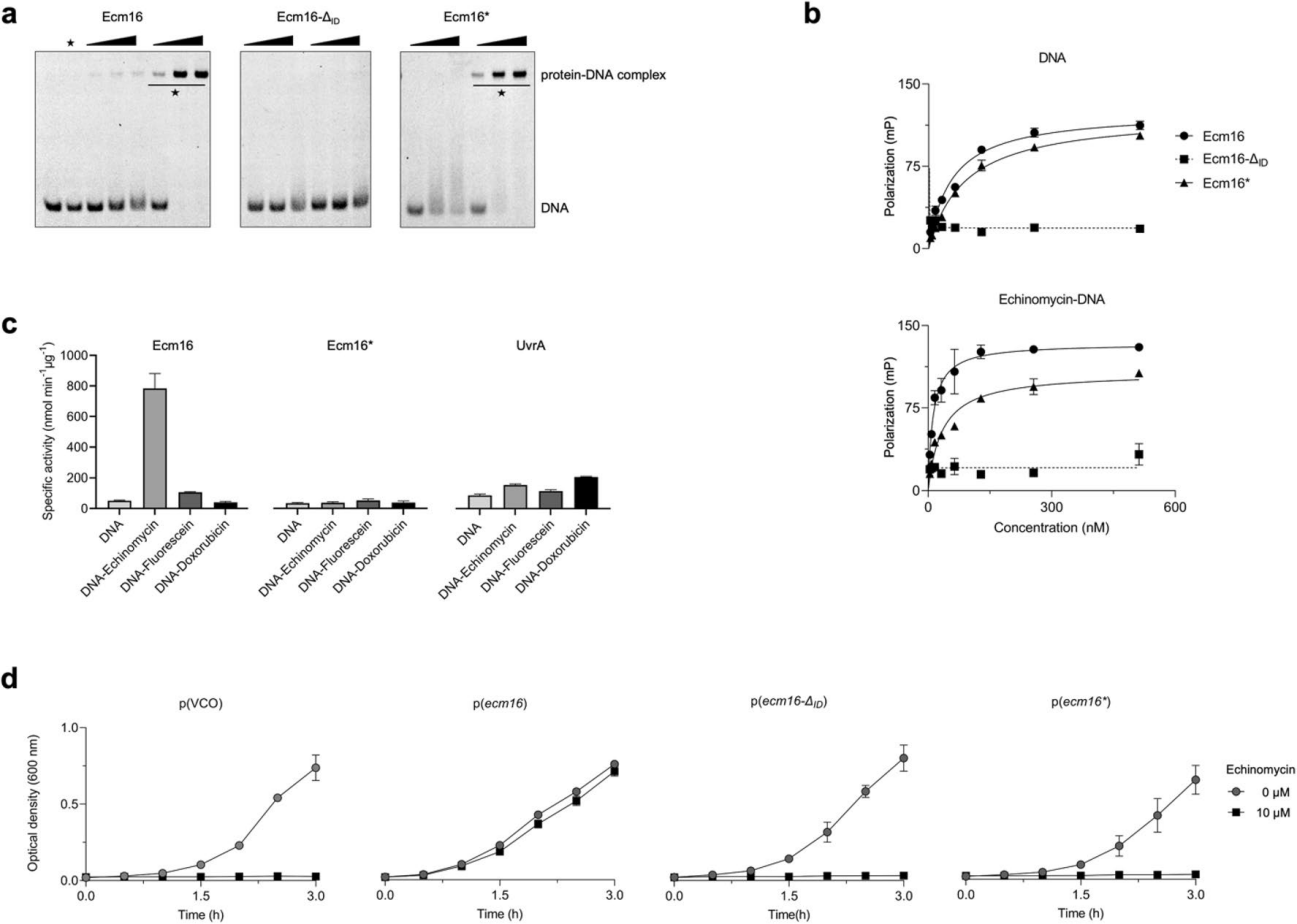
The insertion domain of Ecm16 is required for echinomycin resistance. (**a**) DNA binding activity of wild type and variant Ecm16 proteins analyzed using electrophoretic mobility shift assay. The reaction mixture contained DNA or DNA-echinomycin (marked as asterisk) in the absence (lane 1 and 2) or presence of 100, 200, and 300 nM Ecm16, Ecm16-Δ_ID_ and Ecm16*. Original gels are presented in Supplementary Fig. 9. (**b**) DNA binding activity of wild type and variant Ecm16 proteins analyzed using fluorescence polarization assay. 50 nM fluorescein-labeled 32-bp DNA (top) and 50 nM echinomycin-DNA (bottom) were incubated with increasing amounts of protein. Error bars represent standard deviation of three independent experiments. (**c**) Specific ATP hydrolysis activity of Ecm16, Ecm16*, and UvrA in the presence of 1 µM DNA, DNA-echinomycin, DNA-fluorescein, and DNA-doxorubicin. Error bars represent standard deviation of three independent experiments. (**d**) Determination of echinomycin resistance. Growth curve of *E. coli* (K12) cells carrying vector-control-only, p(VCO), p(*ecm16*), p(*ecm16-Δ*_*ID*_), and p(ecm16*) plasmids in the absence or presence of 10 µM echinomycin. Plots are representative of three independent replicates.

The insertion domain of UvrA and UvrA2 have been proposed to contribute to binding of the DNA substrate^15,20,21^. To test whether the insertion domain of Ecm16 is required for DNA binding, we prepared Ecm16-Δ_ID_ in which the insertion domain was replaced with a glycine-serine linker (Supplementary Figs. 7, 8). We monitored the binding of Ecm16 and Ecm16-Δ_ID_ to a 32-bp DNA that contains a single echinomycin binding site (Supplementary Table 2). Electrophoretic mobility shift assay (EMSA) showed that Ecm16 bound more tightly to echinomycin-bound DNA than normal DNA, whereas Ecm16-Δ_ID_ did not bind to either type of DNA (Fig. 2a). Next, we measured the DNA binding affinity of Ecm16 and Ecm16-Δ_ID_ using fluorescence polarization. The dissociation constant for Ecm16-DNA-echinomycin and Ecm16-DNA was 11.8 nM and 60.2 nM, respectively (Fig. 2b, Table 3). For Ecm16-Δ_ID_, no binding was observed for either DNA substrate. Therefore, EMSA and fluorescence polarization both showed that the insertion domain of Ecm16 is required for DNA binding.

**Table 3:** The apparent K_D_ calculated from fluorescence polarization assay, specific activity, and doubling time measurements of WT Ecm16, Ecm16-Δ_ID_ and Ecm16*.

Protein	K_D_ (nM)^a^		Specific activity (nmol min^−1^ µg^−1^)	*E. coli* doubling time (h)	
	DNA	Echinomycin-DNA		0 µM	10 µM
WT Ecm16	60.2 ± 2.1	11.8 ± 1.7	783.8 ± 97.5	0.56 ± 0.08	0.60 ± 0.10
Ecm16-Δ_ID_	UD	UD	2.9 ± 0.4	0.50 ± 0.03	UD
Ecm16*	90.8 ± 3.3	37.7 ± 1.9	42.9 ± 2.5	0.50 ± 0.01	UD

^a^Values shown are from triplicate experiment. UD, undetectable.

Next, we prepared Ecm16* in which the insertion domain of Ecm16 was exchanged with the insertion domain of DrrC, an Ecm16 homolog from *Streptomyces peucetius* (Supplementary Figs. 7, 8). DrrC was reported to confer resistance against the DNA monointercalator antibiotic daunorubicin, although the molecular mechanism of DrrC is not known^22^. The insertion domain of Ecm16 and DrrC share 32% amino acid sequence identity. Ecm16* bound to echinomycin-containing DNA 2.4-fold more tightly than to normal DNA (K_D_ = 37.7 nM vs. 90.8 nM) (Fig. 2b, Table 3). This result indicates that having a homologous insertion domain is sufficient for Ecm16 to distinguish echinomycin-bound DNA from normal DNA through differential binding. To investigate whether Ecm16* has anti-echinomycin activity, *E. coli* K12 cells expressing Ecm16* were challenged with 10 µM echinomycin. Ecm16* expressing cells were sensitive to echinomycin (Fig. 2d), indicating that the native insertion domain must be present to provide anti-echinomycin activity.

### DNA-echinomycin stimulates the ATP hydrolysis activity of Ecm16

We reported previously that the ATP hydrolysis activity of Ecm16 is required to render echinomycin resistance in vivo^14^. Because Ecm16* bound preferentially to echinomycin-containing DNA yet it failed to confer echinomycin resistance, we predicted that Ecm16* could not hydrolyze ATP. To test this idea, we measured the ATP hydrolysis activity of Ecm16, Ecm16-Δ_ID_, Ecm16*, and UvrA in the presence of drug-free DNA, echinomycin-bound DNA, fluorescein-modified DNA, and doxorubicin-bound DNA (Supplementary Table 2). Echinomycin-bound DNA is the presumed native substrate of Ecm16, fluorescein-modified DNA mimics UV-damaged DNA and is a known substrate for UvrA but not Ecm16^14,20^, and doxorubicin-bound DNA is the presumed substrate for DrrC^23^. The ATP hydrolysis activity of Ecm16 increased by 16-fold in the presence of echinomycin-bound DNA but not other DNA substrates we tested (Fig. 2c). Ecm16* and UvrA showed a similar basal ATPase activity for all four DNA substrates. Ecm16-Δ_ID_ displayed no significant ATP hydrolysis activity for all DNA substrates (Supplementary Fig. 10), which is consistent with the inability of Ecm16Δ_ID_ to bind DNA (Fig. 2a, b). These results indicate that only the native DNA substrate stimulates the ATP hydrolysis activity of Ecm16 and that this property is lost when the insertion domain is deleted or when it is substituted with the insertion domain of a homologous protein. Therefore, the insertion domain plays an important role in determining Ecm16’s substrate specificity and in supporting Ecm16’s anti-echinomycin activity.

### Ecm16 provides resistance against a variety of quinomycin antibiotics

**Figure 3:**
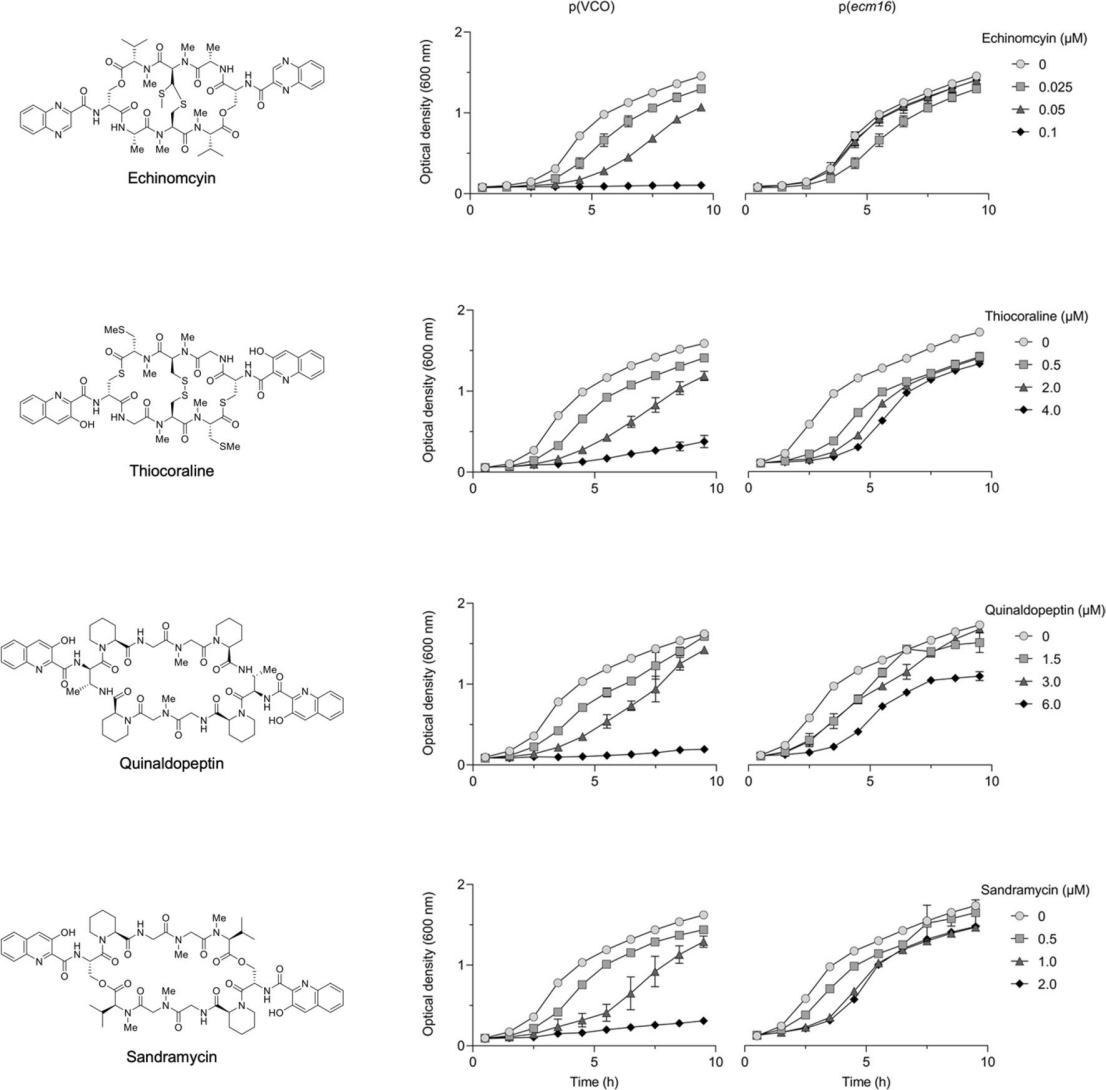
Growth curve study of *B. choshinensis* strains with pNI vector-control-only (VCO) or *ecm16*_pNI plasmid. Cells were grown in 2SY media supplemented with 50 µg/ml neomycin antibiotic. *B. choshinensis* strain was incubated with indicated concentrations of echinomycin (0.025, 0.05, 0.1 µM), thiocoraline (0.5, 2.0, 4.0 µM), quinaldopeptin (1.5, 3.0, 6.0 µM) and sandramycin (0.5, 1.0, 2.0 µM) DNA bis-intercalators. Error bars represent standard deviation of three independent experiments.

We probed the substrate specificity of Ecm16 by testing whether Ecm16 can provide resistance against other DNA-binding drug molecules—doxorubicin, mitomycin C, daunorubicin, actinomycin D, cisplatin, thiocoraline, quinaldopeptin, and sandramycin. Because the permeability of these compounds is limited in Gram negative (diderm) bacteria, we used the Gram positive (monoderm) bacterium *Brevibacillus choshinensis*, instead of *E. coli* K12. Ecm16-expressing *B. choshinensis* cells displayed resistance only against the DNA bisintercalator antibiotics echinomycin, thiocoraline, quinaldopeptin, and sandramycin (Fig. 3). The degree of resistance provided by Ecm16 was most pronounced at the highest antibiotic concentration tested. Cells containing the control vector grew very slowly in the presence of 0.1 µM echinomycin, 4 uM thiocoraline, 6 µM quinaldopeptin, or 2 µM sandramycin making it impossible to determine the doubling time, whereas cells expressing Ecm16 had doubling times which were more similar to cells which were not treated with the respective antibiotic (only 1.1- to 1.2-fold longer) (Fig. 3). Our result showed that Ecm16 is most effective against echinomycin, but it also provides some resistance against other structurally similar quinomycin antibiotics.

## Discussion

Here we report the crystal structure of Ecm16 from the echinomycin producer *S. lasalocidi*. Ecm16 is a homolog of UvrA, the DNA damage sensor protein from the prokaryotic NER pathway. The main structural difference between Ecm16 and UvrA is that Ecm16 lacks the UvrB-binding domain and a zinc-binding module which are present in all UvrA structures reported to date (PDB ID: 2R6F, 3PIH, 3UWX, 3UX8, 3ZQJ, 6N9L). Another potential structural difference is the conformation of the ~ 100 residue insertion domain, although this remains to be verified since the insertion domain is not visible in the Ecm16 crystal structure, presumably because this domain is mobile in the absence of a bound DNA substrate. Overall, the three-dimensional structure of Ecm16 and UvrA are highly similar. They share the same protein fold and they both contain four ATP-binding sites and one continuous DNA-binding groove. Accordingly, Ecm16 and UvrA both display ATPase activity and bind double-stranded DNA^14^. However, Ecm16 lacks the UvrB-binding domain and its associated zinc-binding module, suggesting that Ecm16 and UvrA have distinct molecular mechanisms acquired potentially through divergent evolution.

Ecm16Δ_ID_, which lacks the insertion domain, failed to bind DNA. Furthermore, expression of Ecm16Δ_ID_ in *E. coli* K12 did not protect the cells from echinomycin. Ecm16*, which possesses the insertion domain from the daunorubicin resistance protein DrrC, showed 2.4-fold higher binding affinity to echinomycin-bound DNA than normal DNA. However, Ecm16*, in contrast to Ecm16, did not display the dramatic increase in ATP hydrolysis rate in the presence of echinomycin-containing DNA. Based on these results, we propose a two-step model for detection of echinomycin-bound DNA by Ecm16. In the first step, Ecm16 discriminates echinomycin-bound DNA from normal DNA by differential DNA binding affinity. This initial screening step requires the presence of the insertion domain independent of sequence. In the second step, Ecm16-bound DNA substrates are further discriminated by their ability to stimulate the ATPase activity of Ecm16. This second step appears to require an insertion domain that is specifically matched to echinomycin. Additional structural studies are needed to determine how these two steps are achieved at the molecular level. In particular, atomic structure of Ecm16 in complex with echinomycin-bound DNA and with echinomycin-free DNA will help decipher the molecular mechanism. Interestingly, Ecm16 also provided protection against the natural product DNA bisintercalators thiocoraline, quinaldopeptin, and sandramycin. This is reminiscent of UvrA protein’s ability to detect a wide variety of DNA lesions.

Understanding antibiotic resistance mechanisms is important because it enables the development of new therapeutic strategies. Our work has started to unravel a potentially novel antibiotic resistance mechanism, but further studies are needed to fully understand how Ecm16 confers echinomycin resistance. This includes determining the structure of Ecm16 bound to various DNA substrates and identifying, if any, Ecm16’s partner proteins. Assuming Ecm16 requires partner proteins, they are likely to be proteins which are conserved throughout different phylogenetic lineages since Ecm16 confers echinomycin resistance when expressed in three distantly related organisms, *S. lasalocidi*, *E. coli, and B. choshinensis*.

## Methods

### Bacterial strains, media, and growth conditions

The strains and plasmids used in this study are listed in Table S4. *E. coli* DH5α (Invitrogen) strain was used for cloning, plasmid propagation, while growth curve studies were performed using *E. coli* (K12) (Invitrogen) strain. Luria–Bertani (LB) broth (Difco) with appropriate antibiotic was used to grow *E. coli* cultures at 37 °C with constant aeration and shaking at 200 rpm. *E. coli* strains cultured on solid media were plated on LB agar (Fisher BioReagents) and incubated at 37 °C. 0.2% l-Arabinose (Alfa Aesar) was added for induction of gene expression in LB growth cultures. *B. choshinensis* strain (Takara Bio) was grown in MT (10 mg ml^−1^ glucose, 10 mg ml^−1^ phytone peptone, 35% ehrlich bonito extract, 2 mg ml^−1^ yeast extract blue label, 10 µg ml^−1^ FeSO_4_, 10 µg ml^−1^ MnSO_4_, 1 µg ml^−1^ ZnSO_4_, 4.1 µg ml^−1^ MgCl_2_) media. For growth curve studies, *B. choshinensis* strain was cultured in 2SY (20 mg ml^−1^ glucose, 40 mg ml^−1^ bacto soytone, 5 mg ml^−1^ bacto yeast extract, 150 µg ml^−1^ CaCl_2_) liquid media at 37 °C with shaking at 200 rpm. *E. coli* and *B. choshinensis* cells harboring different plasmids were maintained in presence of ampicillin (50 µg ml^−1^ liquid media and 100 µg ml^−1^ solid media for *E. coli*) and neomycin (50 µg ml^−1^ for *B. choshinensis*). *B. choshinensis* strains were maintained as stock cultures at − 80 °C. Stock cultures were frozen in LB medium containing 40% glycerol.

### Ecm16/Ecm16-Δ_ID_/Ecm16* cloning and protein expression

Codon optimized genes for *ecm16* and *ecm16-Δ*_*ID*_ were subcloned into the pUC19 vector using the NdeI and EcoRI sites (GenScript) for expression in *E. coli*. The genes were digested using enzymes NdeI and EcoRI and gel purified. pET28a (+) expression vector was cut with NdeI and EcoRI and gel purified. The *ecm16* and *ecm16-Δ*_*ID*_ inserts were ligated into the pET28a(+) vector at a 3:1 insert:vector ratio using the quick Ligation Kit (New England Biolabs Inc). Ligation products were transformed into chemically competent *E. coli* DH5α cells and grown overnight on LB-kan plates (50 µg ml^−1^) at 37 °C. *E. coli* BL21 (DE3) (Novagen) cells were transformed with the expression vector and then cultured to exponential phase at 37 °C in Luria Bertani (LB) medium containing 50 µg/ml kanamycin. Ecm16* was cloned into pET28a (+) vector using the same procedure using EcoR1 and HindII restriction digestion enzymes at 5′ and 3′ site respectively. Expression of *ecm16* was induced using 0.25 mM isopropyl-d-1-thiogalactopyranoside (Thermo Scientific) at an optical density at OD_600_ of 0.6–0.8. Cells were further grown for 16 h at 18 °C and then harvested by centrifugation at 6000 × g for 15 min at 4 °C. Cell pellet was resuspended in lysis buffer (50 mM HEPES pH 7.5, 200 mM NaCl, 10 mM imidazole, and 5% glycerol (v/v), 1 mM phenylmethylsulphonyl fluoride, 10 µg/ml DNase and 10 mM MgCl_2_) and lysed by sonication. Cell lysate was centrifuged at 30,000 × g for 60 min at 4 °C and insoluble material was removed. Soluble fraction was loaded on a 5 ml Ni-NTA column (GE Healthcare) that was equilibrated with binding buffer (50 mM HEPES pH 7.5, 200 mM NaCl), washed with 250 ml washing buffer (50 mM HEPES pH 7.5, 200 mM NaCl, 30 mM imidazole, and 5% (v/v) glycerol), and bound protein was eluted with elution buffer (50 mM HEPES pH 7.5, 200 mM NaCl, 250 mM imidazole). Eluted protein solution was applied to a 5 mL HiTrap QHP HP column (GE Healthcare), and bound protein was eluted using a linear 50–500 mM NaCl gradient. Lastly, the Ecm16 samples were purified by size exclusion chromatography in 50 mM HEPES pH 7.5, 50 mM NaCl using a Superdex 200 10/300 GL (GE Healthcare). The purity of protein was assayed by SDS-PAGE. Protein samples were concentrated using Amicon 10-kDa MWCO centrifugal filter. All purification steps were performed at 4 °C. Ecm16-Δ_ID_ and Ecm16* variants were prepared in the same manner as the wild type Ecm16 protein.

For in vivo studies using *E. coli*, codon-optmized *ecm16* and *ecm16-Δ*_ID_ from previously constructed pET28a vectors were amplified with SacI and EcoRI sites using Phusion polymerase (New England Biolabs). The genes were electrophoretically separated on a 0.7% agarose gel and gel purified. Following purification the genes were digested with SacI and EcoRI (New England Biolabs) and cloned into a similarly digested pBAD-Myc-HisA vector (Thermo Fisher). Following digestion, the vector was treated with shrimp alkaline phosphatase (New England Biolabs). Both the digested vector and digested fragments were electrophoretically separated on a 0.7% agarose gel and gel purified again.The fragments were ligated into the pBAD vector using T4 DNA ligase (New England Biolabs), then transformed into electrocompetent DH-5α cells and grown overnight on LB-amp plates (100 µg ml^−1^) at 37 °C. *E. coli* BW25113 (Coli Genetic Stock Center) cells were transformed with the expression vector and then cultured to exponential phase at 37 °C in LB medium containing 50 µg/ml ampilcillin. Construction of BW25113 cells with ecm16* was performed similarly to BW25113-*ecm16* and BW25113-*ecm16-Δ*_*ID*_*,* with the exception that HindIII and EcoRI sites were used for restriction digestion.

### UvrA expression in *E. coli*

UvrA gene from *Thermotoga maritima* was encoded into the pET28a (+) vector and transformed into *E. coli* Rosetta (DE3) pLysS cells. Protein expression was induced using 50 µM isopropyl-ß-d-galactoside (IPTG) and incubated for 5 h at 30 °C. Bacterial cells were resuspended in buffer containing 50 mM Tris pH 8.0, 150 mM NaCl, 2 mM dithiothreitol (DTT), 1 mM phenylmethylsulfonyl fluoride (PMSF) and 5% (v/v) glycerol and sonicated. The lysate was centrifuged at 18,000 rpm for 45 min and the supernatant was applied on His-Trap crude 5 ml column (GE Healthcare). UvrA was eluted with a 200 ml imidazole gradient from 0 to 500 mM concentration. The purified protein was diluted with buffer containing 50 mM Tris pH 8.0, 2 mM DTT, and 5% (v/v) glycerol and applied to 5 mL HiTrap Q HP column (GE Healthcare). UvrA was eluted with 50 ml linear NaCl gradient from 50 mM to 1 M. The protein was further purified by size exclusion chromatography using Superdex 200 10/300 GL (GE Healthcare) equilibrated with buffer containing 50 mM Tris pH 8.0, 200 mM NaCl, 2 mM DTT and 5% (v/v) glycerol. The volume of the collected protein sample was reduced to a final concentration of 10 mg/ml and flash-frozen in liquid nitrogen.

### Ecm16 crystallization

Ecm16 crystals were grown at 18 °C by vapor diffusion using the hanging drop method by mixing 1 µl of protein solution (8 mg ml^−1^ Ecm16, 10 mM MgCl_2_, 1 mM ADP) with 1 µl of equilibration buffer (0.1 M MES pH 6.5, 0.2 M sodium thiocyanate and 12% PEG (w/v) 20 K). Crystals were transferred into the same buffer including 20% v/v ethylene glycol prior to flash-freezing.

### Data collection and structure determination

Initial X-ray diffraction experiments were carried out at the Stanford Synchrotron Radiation Lightsource. The final X-ray diffraction data set was collected at beamline 17-ID-B of the Advanced Photon Source, Argonne National Laboratory and processed using autoPROC^24^. Molecular replacement was carried out using PHASER^25^ and UvrA2 structure (PDB: 2VF7)^15^ as the search model. Structure refinement was performed using PHENIX^26^ and REFMAC^27^. Model building was done using COOT^28^ with alternate sessions of refinement using PHENIX^26^.

### Ecm16 expression in *B. choshinensis*

Codon optimized *ecm16* gene for expression in *B. choshinensis* was synthesized (GenScript) and inserted into pUC19 vector using the BamHI and XbaI sites. pNI, a shuttle vector between *B. choshinensis* and *E. coli*, was purchased from Takara Bio. The pNI vector is under the P2 promoter, which is a portion of 5' sequence upstream of the cell wall protein, which is expressed strongly in *B. choshinensis. ecm16* was inserted into the pNI vector following the ligation protocol described above. Ligation products were transformed into chemically competent *E. coli* DH5α cells and grown overnight on LB-amp (50 µg ml^−1^) plates at 37 °C. *ecm16*_pNI clone was verified by performing restriction enzyme digestion using BamHI and XbaI. Plasmids *ecm16*_pNI or pNI were transformed in *B. choshinensis* using the New Tris-PEG (NTP) method following the manufacturers guidelines (Takara Bio)^29^. Briefly, 100 ng of plasmid mixed with solution A (Takara Bio) was added to the competent *B. choshinensis* cell pellet and incubated for 5 min at room temperature. Next, PEG containing solution B was added, vortexed, and centrifuged. Cell pellet was resuspended in MT media, followed by incubation at 37 °C for 3 h. Cells were then plated on MT agar plates containing neomycin (50 µg ml^−1^) and cultured overnight at 37 °C. 4–5 colonies were inoculated in 3 ml 2SYNm liquid medium and grown at 30 °C for 48 h with orbital shaking at 120 rpm. The cells were centrifuged, and the cell pellets were resuspended in 1× phosphate (100 mM Sodium phosphate at pH 7.0) buffer. Expression of Ecm16 in *B. choshinensis* was confirmed by SDS-PAGE analysis.

### Growth curve studies

Stock solutions of 908 µM echinomycin (MilliporeSigma) and 864 µM thiocoraline (Cayman Chemical) were prepared in methanol. 805 µM quinaldopeptin (Cayman Chemical) and 819 µM sandramycin (Cayman Chemical) stock solutions were prepared in DMSO. For growth curve and biochemical studies, all compounds were diluted using H_2_O. For *E. coli* growth curve experiments, cultures were grown overnight in LB liquid media supplemented with 30 µg ml^−1^ ampicillin at 37 °C and 200 rpm. Saturated culture was induced with a 0.2% arabinose solution for 30 min and used to inoculate 2 ml duplicate replicate samples at 0.02 OD_600_ in 13 mm glass tubes. OD_600_ readings were taken every 30 min for 6 h. For growth profiling of *B. choshinensis* in 2SYNm media, OD_600_ was measured using a multi-well plate reader (Synergy HT, BioTek). Typically, 3 µl of the overnight culture with an OD_600_ of 1.0–1.2 was used to inoculate a well containing 200 µl of fresh 2SYNm medium and growth was monitored every 30 min for 10 h in a 96-well plate.

### Electrophoretic mobility shift assay

PAGE-purified 32-bp DNA substrate (Integrated DNA Technologies) was dissolved in annealing buffer (30 mM HEPES, pH 7.5, 100 mM potassium acetate). This DNA contained the 5′ ACGT 3′ echinomycin binding site (Table S2). Echinomycin-DNA complex was formed by incubating echinomycin and DNA at molar ratio of 1.1: 1. Different concentrations of purified Ecm16, Ecm16-Δ_ID_, Ecm16* (0, 100, 200, and 300 nM) were incubated with 50 nM DNA in the presence or absence of echinomycin in binding buffer (50 mM HEPES, pH 7.5, 50 mM NaCl, 0.1 mg ml^−1^ bovine serum albumin) for 15 min at room temperature. The reaction mixture was separated in a 6% native polyacrylamide gel at 4 °C using 1× TBE (40 mM Tris acetate, 0.5 mM EDTA) as a running buffer for 30 min at 40 mA. The gels were stained using 1× SYBR gold nucleic acid stain in 1× TBE buffer and imaged using an ultraviolet transilluminator (Azure c200).

### Fluorescence polarization assay

Fluorescein labeled 32-bp DNA oligonucleotide (Integrated DNA Technologies) was dissolved in binding buffer (50 mM HEPES, pH 7.5, 50 mM NaCl) and aliquoted to 50 nM final concentration to the reaction well. Purified Ecm16 or Ecm16-Δ_ID_ or Ecm16* in presence or absence of echinomycin was serially diluted in binding buffer and added to each reaction well to final volume of 100 µl at a concentration ranging from 4 to 512 nM. To detect the change in the light polarization of the FAM-labeled DNA, fluorescent measurements were performed in a 384-well format on a black low-volume plate (Corning) using Synergy HT (BioTek) plate reader with excitation and emission wavelengths of 490 nm and 520 nm, respectively. Reported polarization values are the average of three independent experiments. Data was analyzed in Graph Pad Prism 5 using the Hill equation. The calculated dissociation constants (K_D_) are listed in Table 3.

### ATPase activity assay

The ATPase activity of purified proteins was determined using the EnzChek™ phosphate assay kit (Thermo Fisher). In this assay, inorganic phosphate produced from ATP hydrolysis is utilized by purine nucleoside phosphorylase (PNP) to convert 2-amino-6-mercapto-7-methylpurine (MESG) to ribose 1-phosphate and 2-amino-6-mercapto-7-methylpurine. Product formation is followed by measureing absorbance at 360 nm. Prior to the ATPase assay, echinomycin was incubated with 32-bp DNA at 1.1:1 molar ratio for 15 min at room temperature. Purified Ecm16 (0.2 µM) was incubated with 1 µM DNA or echinomycin-DNA complex at various ATP concentrations (0.125, 0.325, 0.75, 1.0, 1.5 and 2.0 µM) in reaction buffer (50 mM HEPES pH 7.5, 50 mM NaCl, 10 mM MgCl_2_). MESG (200 µM) substrate and PNP (500 µM) enzyme were added and UV absorbance at 360 nm was measured every 30 s using a microplate reader (Synergy HT, BioTek) at 22 °C. To convert optical density at 360 nm to the amount of degraded ATP, a calibration curve was constructed by plotting OD_360nm_ with phosphate standards.

## Supplementary Information

Supplementary Information.

## Data Availability

The Ecm16 coordinates and structure factors have been deposited in the Protein Data Bank with the accession code 7SH1.
