# Supplementary material for: Structural and functional analyses of the echinomycin resistance conferring protein Ecm16 from Streptomyces lasalocidi
Source: Sci Rep. 2023 May 17;13:7980. doi: 10.1038/s41598-023-34437-9 (PMC10192343; doi:10.1038/s41598-023-34437-9)
Supplement: Supplementary file 1 — Supplementary Information. [file 41598_2023_34437_MOESM1_ESM.docx]

Supplementary Information for

**Structural and functional analyses of the echinomycin resistance conferring protein Ecm16 from *Streptomyces lasalocidi***

Priyanka Gade^1^, Amanda Erlandson^2^, Anwar Ullah^1^, Xi Chen^3^, Irimpan I. Mathews^4^, Paola E. Mera^2^* and Chu-Young Kim^1,5^*

^1^Department of Chemistry and Biochemistry, The University of Texas at El Paso, El Paso, TX, USA.

^2^Department of Microbiology, School of Molecular and Cellular Biology, University of Illinois Urbana-Champaign, Urbana, IL, USA.

^3^Key Laboratory of Synthetic and Natural Functional Molecule of the Ministry of Education, College of Chemistry and Materials Science, Northwest University, Xi’an, 710127, China.

^4^Stanford Synchrotron Radiation Lightsource, SLAC National Accelerator Laboratory, Menlo Park, CA, USA.

^5^Present address: Department of Biochemistry, School of Molecular and Cellular Biology, University of Illinois Urbana-Champaign, Urbana, IL, USA

*email: pmera@illinois.edu; chuyoung@illinois.edu

The PDF file includes:

Supplementary Figures 1-10

Supplementary Tables 1-5

References

**
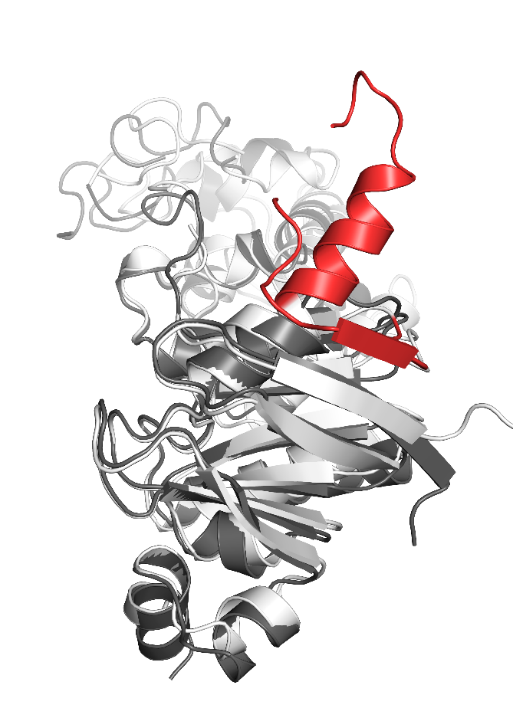
**

**Supplementary Figure 1.** Comparison of NBD-I (white) and NBD-II (grey) of Ecm16. The helix-turn-strand motif (red) is present in NBD-I but not in NBD-II.

**
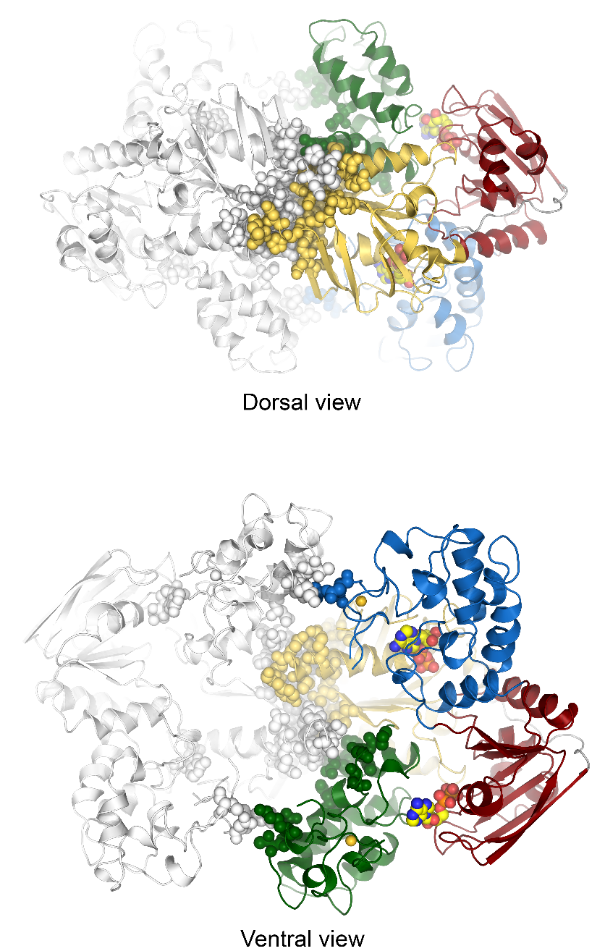
**

**Supplementary Figure 2.** The dimer interface of Ecm16 consists of residues from the ATP-binding I (yellow), signature I (green), and signature II (blue) domains.

**
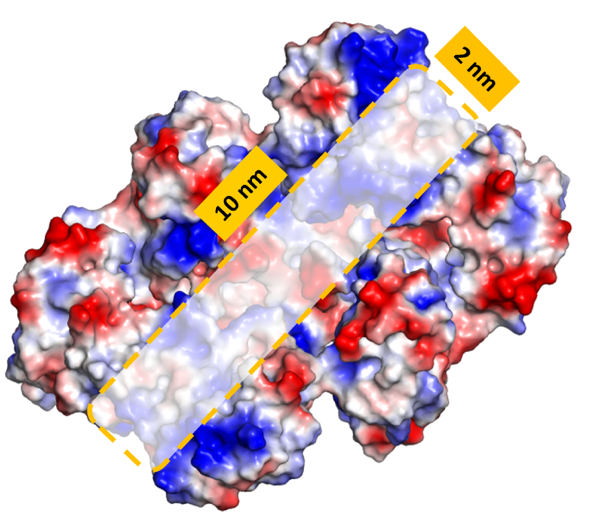
**

**Supplementary Figure 3.** Electrostatic surface potential representation of Ecm16 (red = negative, blue = positive, white = neutral).


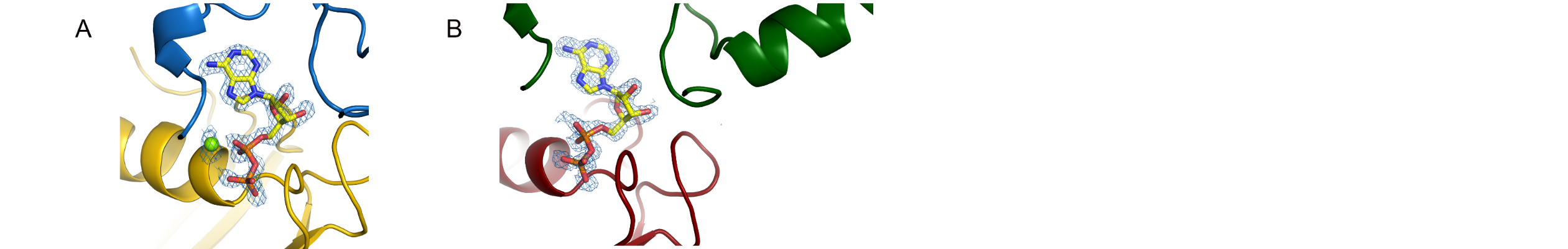


**Supplementary Figure 4.** (A) Proximal and (B) distal nucleotide-binding site of Ecm16. ADP is shown in stick representation and Mg^2+^ ion in the proximal site is shown as a green sphere. Composite omit map of ADP and Mg^2+^ contoured at 2.5 σ.

**
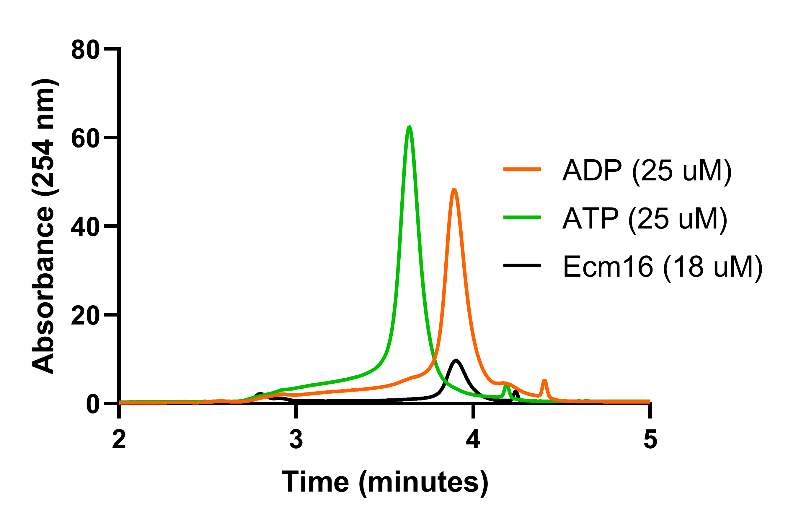
**

**Supplementary Figure 5.** HPLC analysis of nucleotide released from Ecm16 expressed in *E. coli* (black). Pure ATP (green) and ADP (red) dissolved in 50 mM HEPES pH 7.5, 50 mM NaCl were used as standards. All solutions were heated to 65 °C and incubated for 30 minutes prior to analysis.


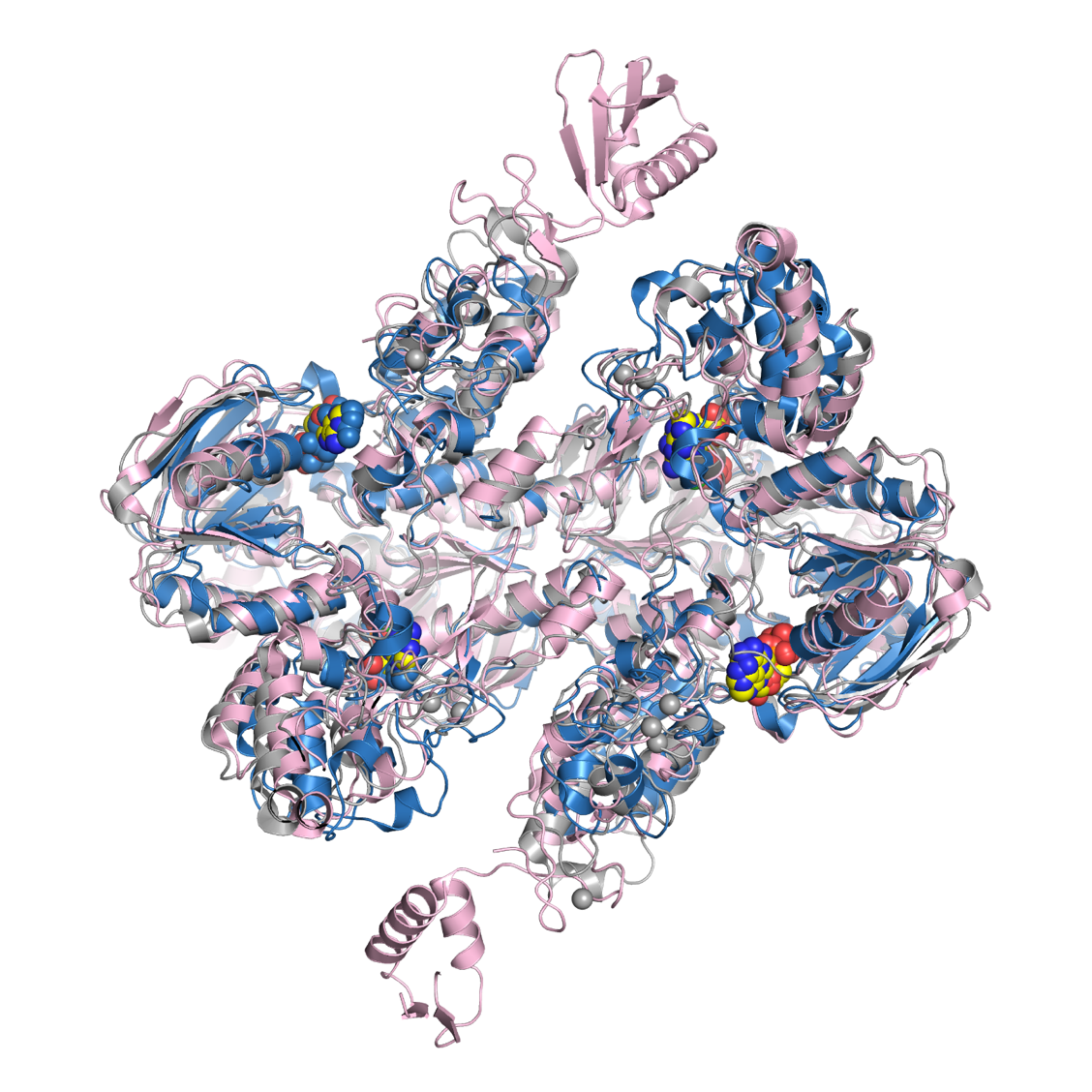


**Supplementary Figure 6.** Superimposition of crystal structures of Ecm16 (PDB ID: 7SH1) (blue) from *Streptomyces lasalocidi*, UvrA (PDB ID: 2R6F) (pink) from *Bacillus stearothermophilus* and UvrA2 (PDB ID: 2VF8) (grey) from *Deinococcus radiodurans*.

**Supplementary Figure 7.** Sequence alignment of Ecm16, Ecm16-Δ_ID_, and Ecm16*. Ecm16-Δ_ID_ contains a GS linker (cyan) in place of the native insertion domain. Ecm16* contains the insertion domain of DrrC (yellow) in place of the native insertion domain.


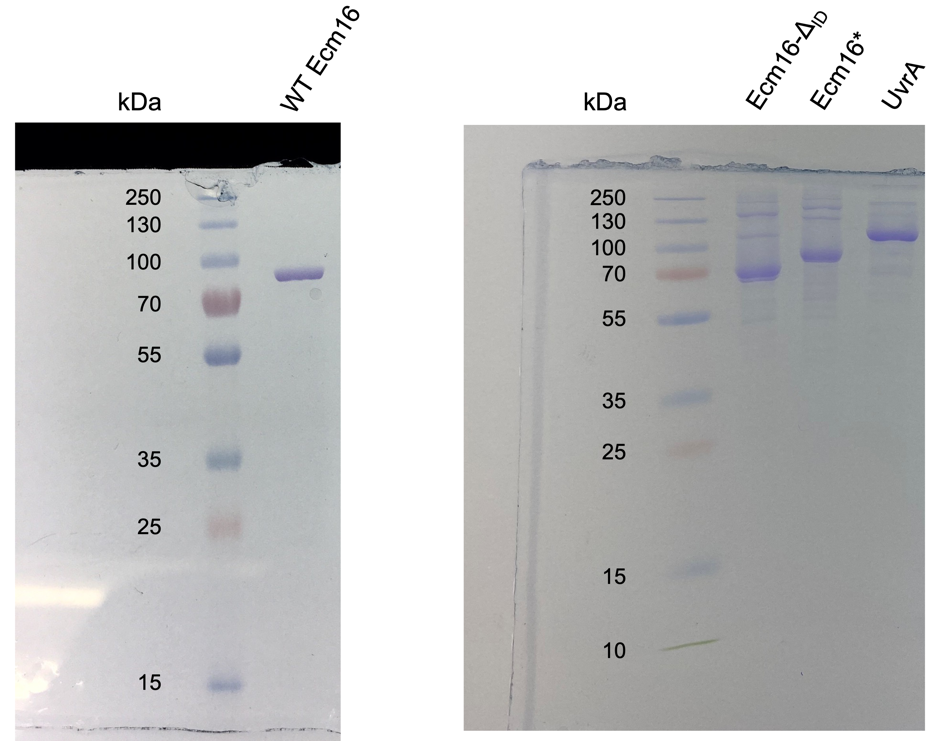


**Supplementary Figure 8.** SDS-PAGE analysis of purified recombinant wild type Ecm16, Ecm16-Δ_ID,_ Ecm16*, and UvrA. Theoretical molecular weight: Ecm16 = 86.3 kDa, Ecm16Δ_ID_ = 72.6 kDa, Ecm16* = 86.1 kDa, and UvrA = 103.9 kDa.


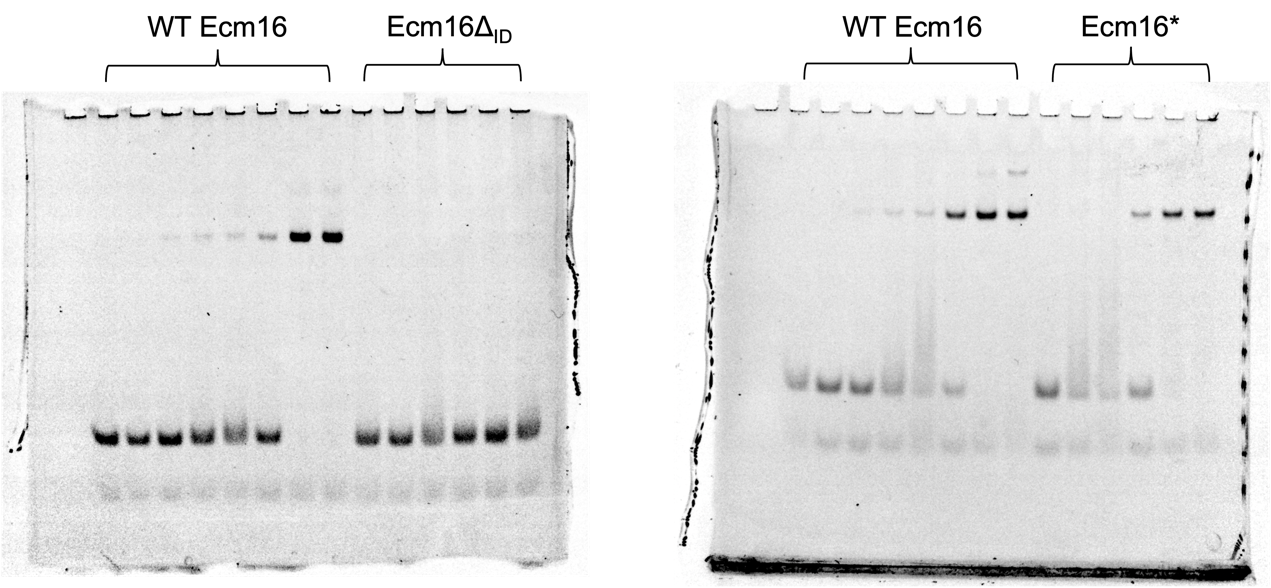


**Supplementary Figure 9.** Original gel pictures of the electrophoretic mobility shift assay result presented in Figure. 2.


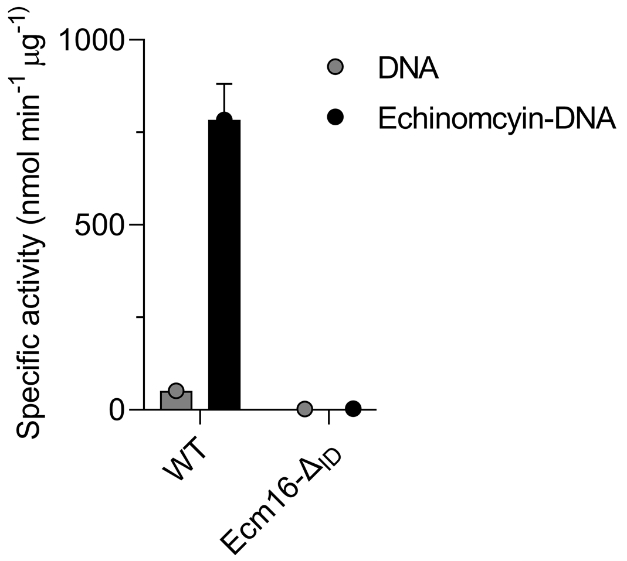


**Supplementary Figure 10.** ATP hydrolysis activity of WT, Ecm16Δ_ID_ in the presence of 1 µM DNA (grey filled circle) or DNA-echinomycin (black filled circle). Error bars represent standard deviation of three independent experiments.

**Supplementary Table 1.** Unmodelled residues in the Ecm16 crystal structure.

| **Chain** | **Residue** | **Domain** |
| --- | --- | --- |
| A | 1-14 | ATP-binding I |
|  | 82-87 | ATP-binding I |
|  | 111-112 | Signature I |
|  | 149-171 | Signature I |
|  | 183-293 | Signature I and insertion |
|  | 599-607 | Signature II |
| B | 1-14 | ATP-binding I |
|  | 79-88 | ATP-binding I |
|  | 152-172 | Signature I |
|  | 185-295 | Signature I and insertion |
|  | 598-607 | Signature II |

**Supplementary Table 2.** DNA substrates used in this study. The echinomycin binding site is highlighted in cyan, and the fluorescein attachment site is highlighted in pink.

| DNA-Echinomycin | 5’-AGTGATCAGTGGTTACGTAACCACTGATCACT-3’  3’-TCACTAGTCACCAATGCATTGGTGACTAGTGA-5’ |
| --- | --- |
| DNA-fluorescein | 5′-AGTGATCAGTGGTTCCGGAACCACTGATCACT-3′  3’-TCACTAGTCACCAAGGCCTTGGTGACTAGTGA-3’ |
| DNA-doxorubicin | 5’-AGTGATCAGTGGTTACGTAACCACTGATCACT-3’  3’-TCACTAGTCACCAATGCATTGGTGACTAGTGA-5’ |
| FAM-DNA-Echinomycin | 5’-FAM-AGTGATCAGTGGTTACGTAACCACTGATCACT-3’  3’-TCACTAGTCACCAATGCATTGGTGACTAGTGA-FAM-5’ |

**Supplementary Table 3.** Specific activities (nmol min^-1^ µg^-1^) of WT Ecm16, Ecm16* and UvrA in presence of DNA, DNA-echinomcyin, DNA-fluorescein, and DNA-doxorubicin.

|  | **Ecm16** | **Ecm16*** | **UvrA** |
| --- | --- | --- | --- |
| DNA | 51.47 ± 0.3 | 34.35 ± 4.3 | 87.87 ± 8.6 |
| DNA-echinomycin | 784.84 ± 97.5 | 42.90 ± 2.5 | 155.25 ± 7.8 |
| DNA-fluorescein | 106.28 ± 4.2 | 52.0 ± 11.0 | 115.55 ± 8.6 |
| DNA-doxorubicin | 40.40 ± 1.7 | 38.70 ± 10.0 | 208.0 ± 12.0 |

**Supplementary Table 4.** E. *coli* strains and plasmids used in this study.

| **Strain #** | **Description** | **Genotype** | **Source** |
| --- | --- | --- | --- |
| BW25111 | BL21 (DE3) | *F^–^ ompT gal dcm lon hsdS_B_(r_B_^–^m_B_^–^) λ(DE3 [lacI lacUV5-T7p07 ind1 sam7 nin5]) [malB^+^]_K-12_(λ^S^)* | Thermo Fisher |
| PM436 | BL21 (DE3)/pET28a-*ecm16* | *F^–^ ompT gal dcm lon hsdS_B_(r_B_^–^m_B_^–^) λ(DE3 [lacI lacUV5-T7p07 ind1 sam7 nin5]) [malB^+^]_K-12_(λ^S^)* / pET28a-*ecm16* | This study |
| PM437 | BL21 (DE3)/pET28a- *ecm16∆_ID_* | *F^–^ ompT gal dcm lon hsdS_B_(r_B_^–^m_B_^–^) λ(DE3 [lacI lacUV5-T7p07 ind1 sam7 nin5]) [malB^+^]_K-12_(λ^S^)* / pET28a- *ecm16∆_ID_* | This study |
| PM438 | BL21 (DE3)/pET28a- *ecm16** | *F^–^ ompT gal dcm lon hsdS_B_(r_B_^–^m_B_^–^) λ(DE3 [lacI lacUV5-T7p07 ind1 sam7 nin5]) [malB^+^]_K-12_(λ^S^)* / pET28a- *ecm16** | This study |
| BW25112 | Rosetta™ 2(DE3)pLysS | Δ(ara-leu)7697 ΔlacX74 ΔphoA PvuII phoR araD139 ahpC galE galK rpsL (DE3) F'[lac^+^ lacI^q^ pro] gor522::Tn10 trxB pLysSRARE (Cam^R^, Str^R^, Tet^R^) | Thermo Fisher |
| PM439 | Rosetta™ 2(DE3)pLysS/pET28a (+)-*uvrA* | Δ(ara-leu)7697 ΔlacX74 ΔphoA PvuII phoR araD139 ahpC galE galK rpsL (DE3) F'[lac^+^ lacI^q^ pro] gor522::Tn10 trxB pLysSRARE (Cam^R^, Str^R^, Tet^R^) / pET28a (+)-*uvrA* | This study |
| BW25113 | K12 parental strain from Keio collection | F-, *Δ(araD-araB)567*, *ΔlacZ4787*(::rrnB-3), *λ*, *rph-1*, *Δ(rhaD-rhaB)568*, *hsdR514* | (2, 3) |
| PM441 | K12/pBAD-Myc-HisA-*ecm16* | F-, Δ(araD-araB)567, ΔlacZ4787(::rrnB3), λ, rph-1, Δ(rhaD-rhaB)568, hsdR514 / pBAD-Myc-HisA-*ecm16* | This study |
| PM601 | K12/pBAD-Myc-HisA-*ecm16∆*_ID_ | F-, *Δ(araD-araB)567*, *ΔlacZ4787*(::rrnB-3), *λ*, *rph-1*, *Δ(rhaD-rhaB)568*, *hsdR514/* pBAD-Myc-HisA-*ecm16* | This study |
| PM619 | K12/pBAD-Myc-HisA-*ecm16** | F-, *Δ(araD-araB)567*, *ΔlacZ4787*(::rrnB-3), *λ*, *rph-1*, *Δ(rhaD-rhaB)568*, *hsdR514/*pBAD-Myc-HisA-*ecm16*/ pBAD-Myc-HisA-*ecm16** | This study |
| BW25114 | *B. choshinensis* | ND^a^ | Takara Bio |
| PM678 | *B. choshinensis*/pNI | ND/pNI | This study |
| PM677 | *B. choshinensis* /pNI-*ecm16* | ND/ *ecm16*-pNI | This study |
| **Plasmids** |  | | **Source** |
| pDNA223 | pET28a-*ecm16* | | This study |
| pDNA269 | pET28a-*ecm16-Δ_ID_* | | This study |
| pDNA281 | pET28a-*ecm16** | | This study |
| pDNA245 | pET28a (+)-*uvrA* | | This study |
| pDNA220 | pBAD-Myc-HisA | | Thermo Fisher |
| pDNA247 | pBAD-Myc-HisA-*ecm16* | | This study |
| pDNA288 | pBAD-Myc-HisA-*ecm16** | | This study |
| pDNA285 | pBAD-Myc-HisA-*ecm16-*∆_ID_ | | This study |
| pDNA289 | pNI | | Takara Bio |
| pDNA290 | pNI-*ecm16* | | This study |
| **Genes** |  | | **Source** |
| Ecm16-5'-SacI-fow | AAAGAGCTCCATATGACCGCGGGTACC | | This study |
| Ecm16-3'-EcoRI-rev | AAAGAATTCTTACGCGCCCACATACTGCG | | This study |
| Ecm16-BamHI-fow | AAAAATCGCGGATCCATGACCGCGGGTACCGAAA | | This study |
| Ecm16-XbaI-rev | AAACGACTAGTCTAGATTACGCGCCCACATACTGCGCC | | This study |
| **Sequencing** |  | | **Source** |
| T7 promoter | TAATACGACTCACTATAGG | | This study |
| T7 terminator | GCTAGTTATTGCTCAGCG | | This study |

^a^The genotype of the *B. choshinensis* strain was not provided by the manufacturer (Takara Bio).

**Supplementary Table 5.** Codon-optimized *ecm16* gene sequences

| ***ecm16* (for expressing in *E. coli*)** |
| --- |
| CATATGACCGCGGGTACCGAAACCGATACCCAGCCGGCGCAACTGTGCGCGGCGGATAGCCACGACATGATTCGTGTTCACGGCGCGCGTGAAAACAACCTGAAAAACGTGCAGGTTGAGATCCCGAAGCGTCGTCTGACCGTTTTCACCGGTGTGAGCGGTAGCGGCAAAAGCAGCCTGGTGTTTGACACCATCGCGGCGGAAAGCCAGCGTCTGATTAACGAAACCTACAGCGCGTTCATCCAAGGTTTTATGCCGACCCTGGCGCGTCCGGAAGTGGATGTTCTGGACGGTCTGACCACCGCGATCCTGGTTGATCAGCAACCGATGGGCACCAGCCTGCGTAGCACCGTGGGTACCGCGACCGATGCGGGTACCCTGCTGCGTATTCTGTTCAGCCGTCTGGCGAAACCGTATATCGGTACCCAGAAGGCGTTCGCGTTTAACGTTGCGAGCGCGGATGCGAGCGGTGTTCTGGTGGTGAACGGTAAGAAAATTGAAAAGGGCTTCAGCGTGGTTGGTGGCATGTGCCTGGCGTGCGAGGGTATCGGCAGCGTTAGCGACATTGATCCGGCGCAACTGTTTGATGCGAGCAAAAGCCTGGCGGATGGTGCGATCACCGTGCCGGGTTGGAAGCCGGATGGTTGGGTGGTTCAGAGCTTCACCGAAAGCGGCTTCTTTGATCCGCACAAAGCGATTCGTGACTACACCGAGCAAGAACGTCACGGCTTTCTGCACGGTGACCCGGTGAAGGTTAAAGTGAAGGGTGTTAACACCACCTATGAAGGCCTGCTGGCGCGTGTGCGTAAGAGCTTTCTGAGCAAGATAAGGAAACCCTGCAGCCGCACATCCGTGCGTTCGTTGACCGTGCGGTGACCTTTAGCGCGTGCAGCGAATGCCACGGTACCCGTCTGAGCGAAACCGCGCGTAGCGCGAAAATCGATGGCCTGAGCATTGCGGACGCGAGCGCGATGCAAATCAGCGATCTGGCGGCGTGGATTCGTGGTCTGACCGACCCGAGCGTTACCACCCTGCTGACCGTGCTGGGTCAGACCCTGGAAAGCTTCGTTCAAATCGGTCTGGGCTACCTGAGCCTGGACCGTAGCAGCAGCACCCTGAGCGGTGGCGAGGCGCAGCGTGTTAAGATGGTGCGTCACCTGGGTAGCGCGCTGACCGATGTTACCTATGTGTTTGATGAACCGACCGTGGGCCTGCACCCGCACGATATTCAACGTATGAACGAGCTGCTGCTGCGTCTGCGTGACAAGGGTAACACCGTTCTGGTGGTTGAGCACAAGCCGGAAACCATCGTGATTGCGGATCATGTGGTTGATCTGGGTCCGCTGGCGGGTACCAAAGGTGGCGAGGTGGTTTTCGAAGGTACCGTTGAAGGTCTGCGTGCGAGCGGTACCGTGACCGGTCGTCACCTGGACGATCGTGCGAGCCTGAAGCCGAGCGTTCGTCAGCGTACCGGTGTGGTTGAGGTGCGTGGCGCGGATGCGCACAACCTGCGTGACGTTGATGTGGACATCCCGCTGGGTGTTCTGACCGTGGTTACCGGCGTGGCGGGTAGCGGTAAAAGCAGCCTGATTCACGGTAGCGTTGCGGGCCGTGATGGTGTGGTTACCGTGGACCAAAGCCCGATCAAGGGTAGCCGTCGTAGCAACCCGGCGACCTACACCGGCATGCTGGAACCGATTCGTAAAACCTTCGCGAAGGCGAACGGTGTTAAACCGGCGCTGTTTAGCCCGAACAGCGAAGGTGCTTGCCCGACCTGCAAGGGTGCGGGCGTTATCTACACCGATCTGGCGATTATGGCGGGTGTGGCGACCACCTGCGAAGACTGCGGTGGCAAACGTTTCCAGCCGAGCGTTCTGCAATATCGTGTGGGTGGCCGTGATATCAGCGAGGTTTTTGCGATGCCGGTTGCGGAGGCGGCGGAGTTCTTCCGTACCGGTGAAGCGCGTACCCCGGCGGCGTGCACCGTTCTGGACCGTCTGGCGGAAGTGGGTCTGGGTTATCTGAGCCTGGGTCAACCGCTGACTACTCTGAGCGGTGGCGAACGTCAACGTCTGAAACTGGCGGGTCACATGGGTGGCGCGGGCAGCGTTTATATCCTGGATGAACCGACCAGCGGTCTGCATCTGGCGGATGTGGAGCAGCTGCTGCGTCTGCTGGATCGTCTGGTTGACAGCGGCAAGACCGTGATTGTGGTTGAGCACCACCAAGCGGTGATGGCGCACGCGGATTGGATCATTGACCTGGGTCCGGGTGCGGGTCATGATGGTGGCCGTGTGGTTTTCGAGGGTACCCCGGCGGATCTGGTTGCGGCGCGTAGCACCCTGACCGGCGAGCACCTGGCGCAGTATGTGGGCGCGTAAGAATTC |
| ***ecm16* (for expressing in *B. choshinensis*)** |
| ATGACAGCAGGCACAGAAACAGATACACAACCGGCGCAGCTTTGCGCAGCGGATTCACATGATATGATCAGAGTGCATGGAGCACGCGAAAATAACCTGAAAAATGTGCAAGTCGAAATTCCGAAAAGACGCCTTACAGTCTTTACAGGCGTTAGCGGCTCTGGAAAATCATCACTGGTCTTTGATACAATTGCTGCCGAAAGCCAAAGACTGATCAACGAAACATACTCTGCTTTTATCCAGGGATTTATGCCGACACTGGCCCGCCCGGAAGTTGATGTGCTGGATGGCCTTACAACAGCTATTCTTGTCGATCAACAGCCGATGGGCACATCACTGAGATCAACAGTTGGAACAGCTACAGATGCCGGCACACTGCTTAGAATTTTATTTTCAAGACTGGCAAAACCGTATATTGGCACACAAAAAGCATTTGCGTTTAATGTTGCTTCTGCCGATGCATCAGGAGTGCTTGTTGTGAACGGCAAGAAAATTGAAAAAGGATTTAGCGTCGTTGGCGGAATGTGCTAGCGTGTGAAGGCATTGGATCAGTCAGCGATATTGATCCGGCGCAGTTATTTGATGCTTCTAAATCACTGGCAGATGGCGCGATTACAGTTCCGGGATGGAAACCGGATGGCTGGGTGGTCCAATCATTTACAGAATCTGGCTTTTTCGATCCGCATAAAGCGATCAGAGATTATACAGAACAGGAAAGACATGGCTTTCTGCATGGCGATCCGGTCAAAGTTAAAGTGAAAGGCGTCAATACAACGTATGAAGGATTACTGGCTAGAGTTCGCAAATCATTTCTTAGCAAAGATAAAGAAACACTGCAACCGCATATCAGAGCCTTTGTGGATCGCGCAGTCACATTTTCTGCGTGCTCAGAATGTCATGGCACAAGACTTTCTGAAACAGCTCGCTCAGCCAAAATTGATGGATTAAGCATCGCGGATGCTTCTGCCATGCAAATTAGCGATCTGGCAGCGTGGATCAGAGGCCTTACAGATCCGAGCGTGACAACACTTTTAACAGTCCTGGGACAAACACTTGAATCTTTTGTTCAGATTGGCCTGGGCTATCTGTCACTGGATCGCTCTTCAAGCACACTTAGCGGCGGAGAAGCTCAAAGAGTGAAAATGGTCCGCCATTTAGGCAGCGCCCTGACAGATGTTACGTATGTTTTTGATGAACCGACAGTGGGATTACATCCGCATGATATTCAGAGAATGAACGAACTGCTTCTGAGATTACGCGATAAAGGAAACACAGTGCTGGTTGTGGAACATAAACCGGAAACAATTGTCATCGCAGATCATGTCGTTGATTTAGGACCGCTGGCGGGCACAAAAGGCGGAGAAGTGGTCTTTGAAGGAACAGTCGAAGGCCTGAGAGCAAGCGGAACAGTTACAGGCAGACATCTGGATGATCGCGCGTCACTTAAACCGAGCGTGAGACAACGCACAGGCGTTGTGGAAGTCAGAGGAGCAGATGCGCATAATCTGCGCGATGTCGATGTTGATATTCCGCTTGGCGTCTTAACAGTCGTTACAGGAGTTGCTGGCTCTGGAAAATCTTCACTTATTCATGGCTCAGTTGCCGGAAGAGATGGCGTGGTCACAGTGGATCAGTCTCCGATTAAAGGCAGCAGACGCTCTAACCCGGCAACATATACAGGAATGTTAGAACCGATCCGCAAAACATTTGCTAAAGCCAATGGCGTTAAACCGGCGCTGTTTTCACCGAACAGCGAAGGAGCTTGCCCGACATGTAAAGGCGCCGGAGTTATTTATACAGATTTAGCAATCATGGCGGGCGTGGCTACAACATGCGAAGATTGTGGCGGAAAAAGATTTCAACCGTCTGTTTTACAGTATAGAGTGGGCGGACGCGATATTTCAGAAGTCTTTGCCATGCCGGTTGCAGAAGCTGCCGAATTTTTCAGAACAGGAGAAGCACGCACACCGGCAGCGTGTACAGTGCTGGATAGACTTGCGGAAGTCGGATTAGGCTATCTGTCACTGGGCCAACCGTTAACAACACTGTCTGGCGGAGAAAGACAGCGCTTAAAACTGGCAGGCCACATGGGCGGCGCAGGATCAGTGTATATTTTAGATGAACCGACAAGCGGCCTTCATTTAGCTGATGTCGAACAACTGCTTAGATTACTGGATCGCCTGGTTGATTCTGGAAAAACAGTGATCGTTGTGGAACATCATCAGGCCGTTATGGCACATGCGGATTGGATTATCGATCTGGGACCTGGAGCTGGACATGATGGCGGAAGAGTCGTTTTTGAAGGCACACCGGCCGATCTTGTTGCTGCCCGCTCAACACTTACAGGCGAACATTTAGCTCAGTATGTGGGAGCC |
| ***ecm16Δ_ID_* (for expressing in *E. coli*)** |
| CATATGACCGCGGGTACCGAAACCGATACCCAGCCGGCGCAACTGTGCGCGGCGGATAGCCACGACATGATTCGTGTTCACGGCGCGCGTGAAAACAACCTGAAAAACGTGCAGGTTGAGATCCCGAAGCGTCGTCTGACCGTTTTCACCGGTGTGAGCGGTAGCGGCAAAAGCAGCCTGGTGTTTGACACCATCGCGGCGGAAAGCCAGCGTCTGATTAACGAAACCTACAGCGCGTTCATCCAAGGTTTTATGCCGACCCTGGCGCGTCCGGAAGTGGATGTTCTGGACGGTCTGACCACCGCGATCCTGGTTGATCAGCAACCGATGGGCACCAGCCTGCGTAGCACCGTGGGTACCGCGACCGATGCGGGTACCCTGCTGCGTATTCTGTTCAGCCGTCTGGCGAAACCGTATATCGGTACCCAGAAGGCGTTCGCGTTTAACGTTGCGAGCGCGGATGCGAGCGGTGTTCTGGTGGTGAACGGTAAGAAAATTGAAAAGGGCTTCAGCGTGGTTGGTGGCATGTGCCTGGCGTGCGAGGGTATCGGCAGCGTTAGCGACATTGATCCGGCGCAACTGTTTGATGCGAGCAAAAGCCTGGCGGATGGTGCGATCACCGCGAAAATCGATGGCCTGAGCATTGCGGACGCGAGCGCGATGCAAATCAGCGATCTGGCGGCGTGGATTCGTGGTCTGACCGACCCGAGCGTTACCACCCTGCTGACCGTGCTGGGTCAGACCCTGGAAAGCTTCGTTCAAATCGGTCTGGGCTACCTGAGCCTGGACCGTAGCAGCAGCACCCTGAGCGGTGGCGAGGCGCAGCGTGTTAAGATGGTGCGTCACCTGGGTAGCGCGCTGACCGATGTTACCTATGTGTTTGATGAACCGACCGTGGGCCTGCACCCGCACGATATTCAACGTATGAACGAGCTGCTGCTGCGTCTGCGTGACAAGGGTAACACCGTTCTGGTGGTTGAGCACAAGCCGGAAACCATCGTGATTGCGGATCATGTGGTTGATCTGGGTCCGCTGGCGGGTACCAAAGGTGGCGAGGTGGTTTTCGAAGGTACCGTTGAAGGTCTGCGTGCGAGCGGTACCGTGACCGGTCGTCACCTGGACGATCGTGCGAGCCTGAAGCCGAGCGTTCGTCAGCGTACCGGTGTGGTTGAGGTGCGTGGCGCGGATGCGCACAACCTGCGTGACGTTGATGTGGACATCCCGCTGGGTGTTCTGACCGTGGTTACCGGCGTGGCGGGTAGCGGTAAAAGCAGCCTGATTCACGGTAGCGTTGCGGGCCGTGATGGTGTGGTTACCGTGGACCAAAGCCCGATCAAGGGTAGCCGTCGTAGCAACCCGGCGACCTACACCGGCATGCTGGAACCGATTCGTAAAACCTTCGCGAAGGCGAACGGTGTTAAACCGGCGCTGTTTAGCCCGAACAGCGAAGGTGCTTGCCCGACCTGCAAGGGTGCGGGCGTTATCTACACCGATCTGGCGATTATGGCGGGTGTGGCGACCACCTGCGAAGACTGCGGTGGCAAACGTTTCCAGCCGAGCGTTCTGCAATATCGTGTGGGTGGCCGTGATATCAGCGAGGTTTTTGCGATGCCGGTTGCGGAGGCGGCGGAGTTCTTCCGTACCGGTGAAGCGCGTACCCCGGCGGCGTGCACCGTTCTGGACCGTCTGGCGGAAGTGGGTCTGGGTTATCTGAGCCTGGGTCAACCGCTGACTACTCTGAGCGGTGGCGAACGTCAACGTCTGAAACTGGCGGGTCACATGGGTGGCGCGGGCAGCGTTTATATCCTGGATGAACCGACCAGCGGTCTGCATCTGGCGGATGTGGAGCAGCTGCTGCGTCTGCTGGATCGTCTGGTTGACAGCGGCAAGACCGTGATTGTGGTTGAGCACCACCAAGCGGTGATGGCGCACGCGGATTGGATCATTGACCTGGGTCCGGGTGCGGGTCATGATGGTGGCCGTGTGGTTTTCGAGGGTACCCCGGCGGATCTGGTTGCGGCGCGTAGCACCCTGACCGGCGAGCACCTGGCGCAGTATGTGGGCGCGTAAGAATTC |
| ***ecm16** (for expressing in *E. coli*)** |
| GAATTCATGACCGCGGGTACCGAAACCGACACCCAGCCGGCGCAACTGTGCGCGGCGGACAGCCACGATATGATTCGTGTGCACGGCGCGCGTGAAAACAACCTGAAGAACGTGCAGGTTGAGATCCCGAAACGTCGTCTGACCGTGTTCACCGGTGTTAGCGGTAGCGGCAAAAGCAGCCTGGTTTTTGATACCATCGCGGCGGAAAGCCAGCGTCTGATTAACGAGACCTACAGCGCGTTCATCCAAGGTTTTATGCCGACCCTGGCGCGTCCGGAAGTGGACGTTCTGGATGGTCTGACCACCGCGATCCTGGTGGACCAGCAACCGATGGGCACCAGCCTGCGTAGCACCGTTGGTACCGCGACCGATGCGGGTACCCTGCTGCGTATTCTGTTCAGCCGTCTGGCGAAGCCGTATATCGGTACCCAGAAAGCGTTCGCGTTTAACGTGGCGAGCGCGGATGCGAGCGGCGTTCTGGTGGTTAACGGTAAGAAAATCGAAAAAGGCTTTAGCGTGGTTGGTGGCATGTGCCTGGCGTGCGAGGGTATCGGCAGCGTGAGCGACATTGATCTGGACAAGCTGCTGGACCGTAGCAAAAGCCTGAACGAAGGTGCGATTCGTCACCCGGCGTTCAACGTTGGTGGCTGGTTTTGGAAGCTGTACGCGAACAGCGGCCTGTTCGATAACGACAAGAAACTGCGTGATTACACCGAGAGCGAATGGCAGGCGTTTCTGTATGGCGCGCAAGGTAGCATCGCGCTGGAATGGCAGGGTGGCAAGGTGAACAGCAAATACGAGGGTCTGATGGACAAGTTCAACCGTCTGTATCTGCGTAAAGAGCCGGATGAAATGAGCGCGAAGAACCGTGTTGCGCTGCAGCAAGTGGTTACCTTTAGCGCGTGCAGCGAATGCCATGGTACCCGTCTGAGCGAGACCGCGCGTAGCGCGAAAATCGACGGCCTGAGCATTGCGGATGCGAGCGCGATGCAAATCAGCGACCTGGCGGCGTGGATTCGTGGTCTGACCGATCCGAGCGTGACCACCCTGCTGACCGTTCTGGGCCAGACCCTGGAGAGCTTCGTGCAAATCGGTCTGGGCTACCTGAGCCTGGACCGTAGCAGCAGCACCCTGAGCGGTGGCGAGGCGCAGCGTGTGAAGATGGTTCGTCACCTGGGTAGCGCGCTGACCGACGTGACCTATGTTTTTGATGAACCGACCGTTGGCCTGCACCCGCACGACATTCAACGTATGAACGAGCTGCTGCTGCGTCTGCGTGATAAGGGTAACACCGTTCTGGTGGTGGAGCACAAGCCGGAAACCATCGTTATTGCGGATCATGTGGTTGATCTGGGTCCGCTGGCGGGTACCAAAGGTGGCGAGGTGGTTTTCGAAGGTACCGTGGAAGGTCTGCGTGCGAGCGGTACCGTTACCGGTCGTCACCTGGACGATCGTGCGAGCCTGAAGCCGAGCGTGCGCCAGCGTACCGGTGTGGTTGAGGTTCGTGGCGCGGATGCGCACAACCTGCGTGATGTGGACGTTGATATCCCGCTGGGTGTGCTGACCGTGGTTACCGGCGTTGCGGGCAGCGGTAAAAGCAGCCTGATTCATGGTAGCGTGGCGGGTCGTGATGGTGTGGTTACCGTTGATCAAAGCCCGATCAAGGGTAGCCGTCGTAGCAACCCGGCGACCTACACCGGCATGCTGGAACCGATTCGTAAGACCTTCGCGAAAGCGAACGGTGTGAAACCGGCGCTGTTTAGCCCGAACAGCGAAGGTGCTTGCCCGACCTGCAAGGGTGCGGGCGTGATCTACACCGACCTGGCGATTATGGCGGGTGTTGCGACCACCTGCGAAGATTGCGGTGGCAAGCGTTTCCAGCCGAGCGTGCTGCAATATCGTGTTGGTGGCCGTGACATTAGCGAGGTGTTTGCGATGCCGGTTGCGGAGGCGGCGGAGTTCTTCCGTACCGGTGAAGCGCGTACCCCGGCGGCGTGCACCGTGCTGGATCGTCTGGCGGAAGTGGGCCTGGGTTACCTGAGCCTGGGTCAACCGCTGACTACTCTGAGCGGTGGCGAACGTCAACGTCTGAAACTGGCGGGTCACATGGGTGGCGCGGGCAGCGTGTATATCCTGGACGAACCGACCAGCGGTCTGCACCTGGCGGATGTTGAGCAGCTGCTGCGTCTGCTGGACCGTCTGGTGGATAGCGGCAAGACCGTTATTGTGGTTGAGCACCACCAAGCGGTTATGGCGCACGCGGACTGGATCATTGATCTGGGTCCGGGTGCGGGTCATGATGGTGGCCGTGTGGTTTTCGAGGGTACCCCGGCGGATCTGGTGGCGGCGCGTAGCACCCTGACCGGCGAGCACCTGGCGCAGTATGTTGGCGCGTAAGCTT |

**References**

1. Cuesta-Seijo JA, Sheldrick GM. 2005. Structures of complexes between echinomycin and duplex DNA. Acta Crystallographica Section D: Biological Crystallography 61:442-448.

2. Baba T, Ara T, Hasegawa M, Takai Y, Okumura Y, Baba M, Datsenko KA, Tomita M, Wanner BL, Mori H. 2006. Construction of *Escherichia coli* K-12 in-frame, single-gene knockout mutants: the Keio collection. Molecular systems biology 2:2006.0008-2006.0008.

3. Datsenko KA, Wanner BL. 2000. One-step inactivation of chromosomal genes in *Escherichia coli* K-12 using PCR products. Proc Natl Acad Sci U S A 97:6640-5.

4. VanDrisse CM, Escalante-Semerena JC. 2016. New high-cloning-efficiency vectors for complementation studies and recombinant protein overproduction in *Escherichia coli* and *Salmonella enterica*. Plasmid 86:1-6.
